# Decrease in HDL-C is Associated with Age and Household Income in Adults from the Korean National Health and Nutrition Examination Survey 2017: Correlation Analysis of Low HDL-C and Poverty †

**DOI:** 10.3390/ijerph16183329

**Published:** 2019-09-10

**Authors:** Kyung-Hyun Cho, Hye-Jeong Park, Suk-Jeong Kim, Jae-Ryong Kim

**Affiliations:** 1 LipoLab, Yeungnam University, Gyeongsan 712-749, Korea; 2 RayDel Lipoprotein Research Center, Daegu 41061, Korea; 3 Department of Biochemistry and Molecular Biology, Smart-Aging Convergence Research Center, College of Medicine, Yeungnam University, Daegu 705-717, Korea

**Keywords:** high-density lipoproteins, cholesterol, house-hold income, dyslipidemia, dementia

## Abstract

A low serum high-density lipoproteins-cholesterol (HDL-C) level is a risk factor of cardiovascular disease and dementia. On the other hand, no study has elucidated the correlation between household income and the HDL-C level in the adult population. In the present study, 5535 subjects (20–80 year-old individuals) were selected from the Korean national health and nutrition examination survey 2017 (KNHANES VII-2, *n* = 2469 men, *n* = 3066 women). They were classified into five levels of household income grades ranging from one (the lowest) to five (the highest). They were also classified according to the HDL-C level: category 1 (<40 mg/dL, *n* = 943), category 2 (40–49 mg/dL, *n* = 1764), category 3 (50–59 mg/dL, *n* = 1572), category 4 (60–69 mg/dL, *n* = 820), and category 5 (≥70 mg/dL, *n* = 436). Generally, in both genders, a higher HDL-C level is associated with a larger percentage of income grades 4 and 5. Moreover, the lowest HDL-C group showed the largest percentage of income grade 1. In both groups, a significant increase in the average income grade was associated with a concomitant increase in the HDL-C level (men, *p* = 0.03, women, *p* < 0.001). In the low HDL-C category, a lower income grade is associated directly with a lower HDL-C level, which suggests that poverty is associated directly with a low HDL-C. Women showed a 3.3-fold higher incidence of dementia than men did at later-life. The sharp decrease in HDL-C in the female group older than 50 was accompanied by a dramatic increase in the incidence of dementia. However, the male group showed a relatively mild decrease in the HDL-C level after mid-life and weak elevation in the incidence of dementia. In conclusion, in both genders, the lower income group showed a larger prevalence of low-HDL-C levels. The decrease in HDL-C after middle age was strongly associated with the considerable increase in dementia in later-life.

## 1. Introduction

Low high-density lipoproteins-cholesterol (HDL-C) levels are a major risk factor of metabolic syndrome [1], mild cognitive impairment (MCI) [2], and dementia [3]. Low HDL-C levels and/or impaired functionality of high-density lipoproteins (HDL) are the hallmarks of dyslipidemia, which is a well-known manifestation of diabetes, cardiovascular disease, and brain disease, including stroke [4]. The prevalence of low HDL-C levels is increasing gradually in developing and developed countries, such as the USA [5] and the Republic of Korea [6]. On the other hand, the prevalence of low HDL-C is increasing more rapidly in developed countries due to a change to a Westernized diet and a lack of exercise. A lower socioeconomic status is also associated with a higher incidence of metabolic syndrome and dementia via the low serum HDL-C level [7]. Frequently, cardiovascular vascular disease (CVD) is associated with the coincidence of MCI and Alzheimer’s disease (AD) [8], even though the pathologic link between CVD and AD remain to be elucidated. As well as prevention of CVD, raising the HDL-C level is a good strategy to attenuate dementia, because HDL can inhibit the aggregation of beta-amyloid (Aβ) and promote the removal of amyloid [9]. Furthermore, HDL reduces the level of vascular Aβ accumulation and attenuates Aβ-induced endothelial inflammation [10,11].

Since HDL can protect low-density lipoproteins (LDL) from oxidation and glycation, the maintenance of HDL in optimal quantity and quality is very important for suppressing the incidence of aging-related diseases, such as coronary heart disease and dementia. A low HDL-C level is frequently associated with an impairment of HDL functionality [12], such as loss of cholesterol efflux, anti-oxidant, and anti-inflammatory activities. Subjects with low HDL-C showed a higher incidence of type II diabetes mellitus (T2DM) than those with a normal HDL-C level. A Canadian study reported that a high HDL-C level is associated with a low prevalence of type 2 diabetes mellitus (T2DM) and obesity [13]. Furthermore, the prevalence of cardiovascular disease (CVD) is closely associated with poverty worldwide. Low neighborhood income and education are independent predictors of incident CVD [14]. Populations with a low socioeconomic status have a higher risk of metabolic syndrome [15], cognitive impairment, and dementia via low HDL-C levels and a loss of HDL functionality. In particular, post-menopausal women have a higher cardiovascular risk [16] and cerebrovascular risk [17] with a rapid decrease of estrogen and HDL-C level. The Korean national health and nutrition examination survey (KNHANES 1998-2005) revealed that Korean men and women have a higher prevalence of low-HDL-C levels with increased obesity in all ages. Between KNHANES 1998 and 2005, the mean HDL-C levels in men and women decreased significantly from 48.1 to 42.3 mg/dL and from 51.6 to 47.1 mg/dL, respectively [6], despite the economic growth in the same period. On the other hand, there are no reports of the correlations between the HDL-C level and household income status in the Korean population. This study analyzed the correlations of age, gender, HDL-C level, and household income from 2017 KNHANES and compared the incidence of dementia in 2017 from Health Insurance Review & Assessment Service in Korea. The aim was to provide statistical evidence for the logical relationships among HDL-C, household income, and incidence of dementia depending on age and gender. 

## 2. Methods

### 2.1. Study Population

The research was based on the Seventh Korean National Health and Nutrition Examination Survey in 2017 (KNHANES VII-2, approval number 117002) performed by the Korea Centers for Disease Control and Prevention. The KNHANES is a nationwide, population-based, cross-sectional survey conducted by the Division of Chronic Disease Surveillance of the Korea Centers for Disease Control and Prevention to examine the health and nutritional status of the population [18]. Trained interviewers collected all data using structured questionnaires and obtained data regarding the socioeconomic status, health, lifestyle, and laboratory information, as well as male and female reproductive history. 

**Figure 1:**
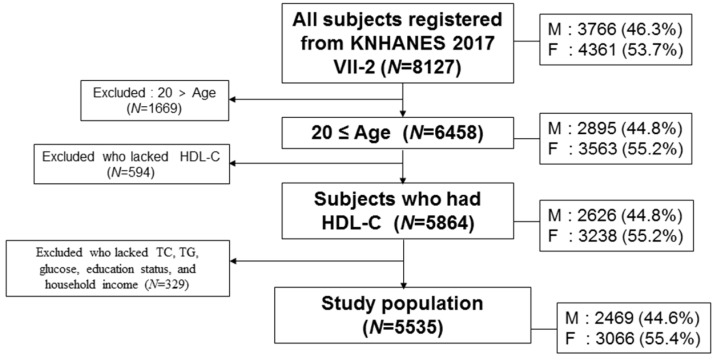
Inclusion criteria and subject number in analysis. HDL-C, high-density lipoprotein cholesterol. TC, total cholesterol. TG, triglycerides. M: men. F: Female.

In the health examination survey, the initial number of subjects in the KNHANES VII-2 (N = 8127) from 3580 households were examined by laboratory measurements for serum cholesterol and questionnaires on health and nutrition. The subjects included in this study were community-dwelling individuals. The total participants in this survey were 5535 individuals (2469 men and 3066 women) based on HDL-C and household income, as shown in Figure 1, who was sampled randomly throughout South Korea. Adult subjects, who were 20–80 years old, reported their household income and blood biochemistry data including total cholesterol (TC), HDL-C, and triglyceride (TG). A low HDL-C level in men and women was defined as <40 and <50 mg/dL, respectively, according to the guidelines of the National Cholesterol Education Program-Adult Treatment Panel III [19].

Self-reported questionnaires were given to the subjects to determine the household income, residential area, marital status, education level, occupation, economic status, smoking, alcohol intake, and physical activity. The household income quintile was divided by the Korean Government based on the subjects’ personal monthly income, which was adjusted based on the average age and gender. Monthly household income was divided according to the range of monthly total income for all family members. The definition of household income was divided into the following and website link (https://knhanes.cdc.go.kr/knhanes/eng/index.do). Monthly household income grade 1 (<595 USD), grade 2 (595–1220 USD), grade 3 (1221–1943 USD), grade 4 (1994–2916 USD), and grade 5 (>2916 USD). In this study, we divided the HDL-C level group into five categories for convenience of comparison from less than 40 mg/dL as a low borderline for the male group [19]: category 1 (<40 mg/dL), category 2 (40–49 mg/dL), category 3 (50–59 mg/dL), category 4 (60–69 mg/dL), and category 5 (≥70 mg/dL), as shown in Table 1.

The serum total cholesterol (TC), HDL-C, and LDL-C were measured directly by a homogeneous enzymatic method using Pureauto SCHO-N, Cholest N HDL, and Cholestest LDL agent (Sekisui, Japan), respectively, with a Hitachi Automatic Analyzer 7600-210 (Hitachi, Japan). The LDL-C was not calculated using the Freidwald formula.

The number of dementia patients in 2017 was provided by the Health Insurance Review & Assessment Service in Korea (Seoul, Korea) and Health Bigdata Hub (https://opendata.hira.or.kr/home.do).

### 2.2. Statistical Analysis

All values are expressed as the mean ± SD (standard deviation) for the continuous variables and as each gender (percentages) for the HDL-C categories and household income grade, which is listed in Table 1 and Table 2. All analyses were normalized by a homogeneity test of variances through Levene’s statistics. If not normalized, nonparametric statistics were used, such as Jonckheere-Terpstra test, Kruskal-Wallis test, and Spearman’s correlation analysis. 

**Figure 2:**
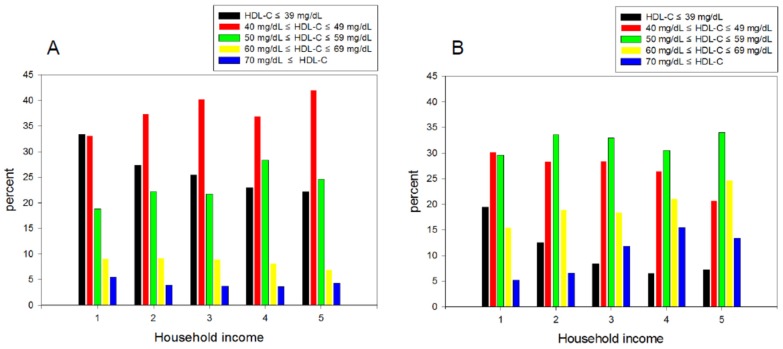
Distribution of high-density lipoprotein cholesterol (HDL-C) category percentage according to household income grade quintile in men (**A**) and women (**B**): KNHANES 2017. Category 1, HDL-C ≤ 39 mg/dL. Category 2, 40 mg/dL ≤ HDL-C ≤49 mg/dL. Category 3, 50 mg/dL ≤ HDL-C ≤ 59 mg/dL. Category 4, 60 mg/dL ≤ HDL-C ≤ 69 mg/dL. Category 5, 70 mg/dL ≤ HDL-C.

**Figure 3:**
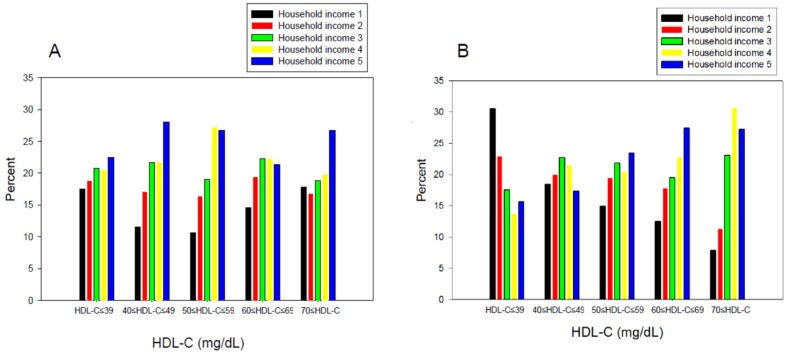
Distribution of household income grade percentage, according to the high-density lipoprotein cholesterol (HDL-C) category in men (**A**) and women (**B**): KNHANES 2017.

The continuous levels of lipid parameters, such as TC, triglyceride (TG), HDL-C, and glucose, were compared using a t-test within each gender. For subcategory analysis, each category of the HDL-C level and household income grade were compared using a t-test depending on gender (Table 1). Table 2 lists the distribution of age and income grade depending on the HDL-C levels. A χ-square test was used to detect the differences in the proportions across household income and HDL-C, respectively (Figure 2 and Figure 3). 

**Figure 4:**
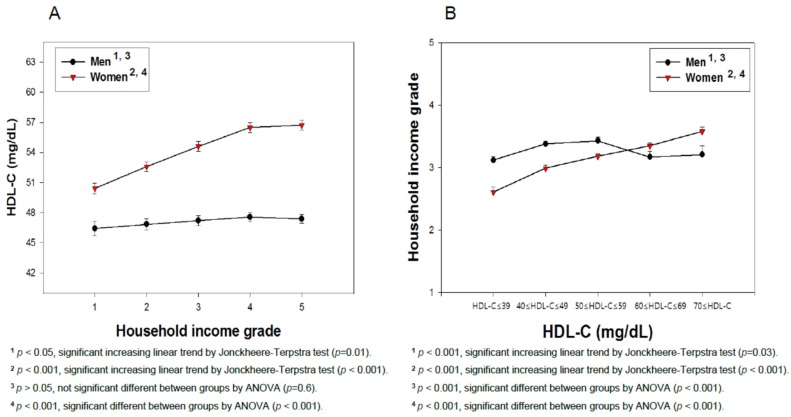
Mean high-density lipoprotein cholesterol (HDL-C) levels according to the household income grade in men and women (**A**). The mean household income grade levels according to the high-density lipoprotein cholesterol (HDL-C) category in men and women (**B**). KNHANES 2017. The data are expressed as the mean ± SEM (Standard Error of the Mean).

**Figure 5:**
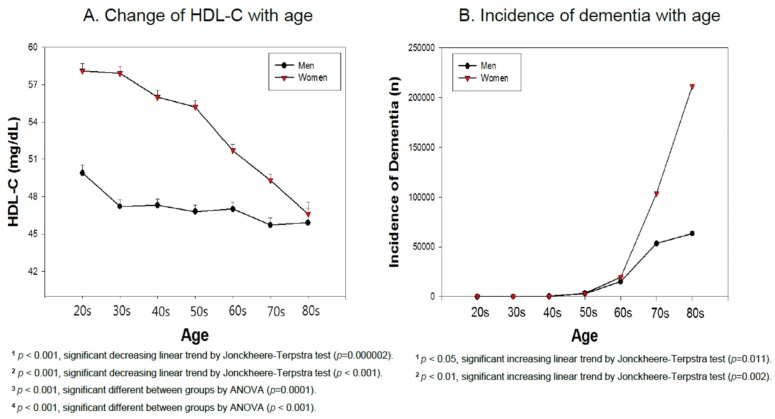
(**A**). Mean high-density lipoprotein cholesterol (HDL-C) levels according to age in men and women from KNHANES 2017. (**B**). Incidence of dementia according to age in men and women in 2017. The data are the mean ± SEM (Standard Error of the Mean).

The differences in the serum concentrations of HDL-C and household income grade depending on gender were determined using ANOVA. The least significant difference (LSD) and Games-Howell post hoc test was used to determine the significance of the differences in the continuous variables to identify the differences between each category (Figure 4 and Figure 5). A multiple group test procedure was performed to determine the pattern, with either an increase or decrease in household income and HDL-C level (Figure 4 and Figure 5), according to Jonckheere-Terpstra (J-T test), as described previously [20,21]. 

Regression analysis was performed to find the correlation between the HDL-C level in each gender and the household income grade (Supplementary Figures S1 and S2). The significance of either an increase or decrease in the HDL-C level in each gender depending on either the household income grade or age was calculated (Supplementary Figures S1 and S2). The relationships between the household income grade and HDL-C levels were estimated using analysis of covariance (ANCOVA) after adjusting for the confounding variables (gender, frequency of diabetes, use of anti-dyslipidemic drugs, myocardial infarction or angina pectoris, physical exercise, alcohol intakes, and smoking habit), as shown in Supplementary Table S1 and Supplementary Figure S3. All analyses were normalized by a homogeneity test of variances through Levene’s statistics.

All tests were two-tailed and the statistical significance was defined at *p* < 0.05. Statistical analyses were carried out using the SPSS statistical package version 25.0 (SPSS Inc., Chicago, IL, USA) incorporating sampling weights and adjusting for the complex survey design of the KNHANES 2017. 

### 2.3. Ethics Statement

Korea National Health and Nutrition Examination Survey (KNHANES) has been an annual review and has been approved by KCDC Research Ethics Review Committee since 2007 (approval no. 2013–12EXP03–5C). The committee operates under the KCDC Research Ethics Review Committee’ s standard guidelines based on domestic & international regulations and guidelines, such as the Declaration of Helsinki and the Bioethics and Safety Act. Informed consent was obtained from all participants when the surveys were conducted.

## 3. Results

### 3.1. Baseline Characteristics

A total of 5,535 subjects (average 51.6 ± 16.4 year-old individuals) showed a normal lipid profile with 193.3 ± 38.1 mg/dL of TC and 133.6 ± 103.4 mg/dL of TG, as shown in Table 1. The male group showed a 1.4-fold higher serum TG level than the female group (*p* < 0.001), whereas the female group showed a higher serum TC level than the male group even though the TC level was within the normal range. The serum glucose levels of both genders were within borderline of the normal range around 110 mg/dL. The LDL-C level was similar between men (118.7 ± 37.6 mg/dL, *N* = 548) and women (122.0 ± 39.4, *N* = 274). They had a normal LDL-C level (119.8 ± 38.2) (*N* = 822). The male group showed a slightly higher average household income grade (3.30 ± 1.4) than the female group (3.15 ± 1.4, *p* = 0.00006). 

In men, the average HDL-C level was 47.2 mg/dL, which ranged from 17 to 114 mg/dL. The percentage distribution of the HDL-C category based on the income level showed a significant correlation (*p* = 0.009), according to χ-square analysis (Gamma correlation coefficient 0.047). In the female group, the mean HDL-C level was 54.4 mg/dL, which ranged from 20 to 120 mg/dL. The percentage distribution of the HDL-C category based on the income level showed a significant correlation (*p* < 0.001), according to χ-square analysis (Gamma correlation coefficient 0.183) in the female group.

In the highest HDL-C category (>70 mg/dL), the mean age of the men and women was 49.5 ± 17.6 and 45.8 ± 14.0 years, respectively (Table 1). In contrast, in the lowest HDL-C category (<40 mg/dL), the male and female groups showed a mean age of 54.3 ± 15.8 and 61.3 ± 14.9 year, respectively, which suggests that an older age is associated with a lower HDL-C level. The female group showed a larger age gap (approximately 15.5 years) between the low and high HDL-C level, whereas the male group showed a gap of 4.8 years. This suggests that the female group showed a more sensitive change in the HDL-C level depending on an increase of age.

The percentage of HDL-C in TC was 25.5 ± 7.1 and 28.6 ± 7.2% for men and women, respectively, which represents a normal level for ordinary subjects. Both groups showed a similar distribution of income grade depending on age. A higher age is associated with a lower income grade. In the highest income category, grade 5, men and women were aged 46.6 ± 14.0 and 46.1 ± 13.2 years, respectively. In the lowest income quintile, grade 1, the men and women were aged 63.2 ± 17.0 and 67.9 ± 12.7 years, respectively. The female group showed a larger age gap (approximately 21.8 years) between the low and high end of the income grade, whereas the male group showed a gap of 16.6 years. Generally, both the HDL-C level and household income grade are negatively associated with the mean age of each category. Correlation analysis revealed a positive association between the HDL-C level and household income in both groups, but the female group (r = 0.164, *p* < 0.001) showed a steeper and more significant association than the male group (r = 0.026, *p* = 0.173), as shown in Supplementary Figure S1. The lowest income grade showed the lowest HDL-C level in both men and women, which suggests that the income grade is associated directly with the HDL-C levels in both groups. The female group showed clearer correlation in that a lower HDL-C level is associated directly with a lower income than those of the male group (Supplementary Figure S1). 

### 3.2. Distribution of Age and Income Grade Depends on HDL-C

Both men and women showed a similar pattern of age and HDL-C distribution. Both groups showed an inverse relationship between age and the HDL-C level (Table 2). Those in their fifties and twenties comprised the largest percentage (20.2%) and smallest percentage (11.3%), respectively. Men and women in their twenties showed the highest HDL-C level of 49.9 ± 11.1 and 58.1 ± 11.3 mg/dL, respectively, whereas those in their seventies showed the lowest HDL-C level of 45.7 ± 11.6 and 49.3 ± 11.4, respectively. These results clearly show that the decrease in HDL-C level with age is larger and faster in women than men. The male group showed a 4.2 mg/dL reduction (8.5% reduction, *p* = 0.000002) from their twenties to seventies, whereas the female group showed an 8.8 mg/dL reduction (15.2% reduction, *p* < 0.001). The male group showed only a 1 mg/dL difference between income grades 1 and 5, even though there was no significance (*p* = 0.237), whereas the female group showed a 6.3 mg/dL difference (*p* < 0.001) between income grades 1 and 5 (Table 2). These results suggest that women exhibited a larger and significant gap of the HDL-C level based on their age or income grade. 

Regression analysis revealed a weak inverse association between age and HDL-C for the male group (r = −0.085, *p* = 0.00001), as shown in Supplementary Figure S2A. Regression analysis revealed a weak inverse association between age and HDL-C (r = −0.240, *p* < 0.001) in the female group. The total number of subjects showed inverse associations between the HDL-C level and age, i.e., they showed a declining pattern of HDL-C with age (r = −0.161, *p* < 0.001).

### 3.3. Percentage Ratio of the HDL-C Level in Each Income Category

The male and female group showed a slightly different distribution of the percentage of HDL-C category, particularly in the lowest and highest income quintile. In men and women, the lowest HDL-C category (<39 mg/dL) was dominant in the lowest income group, as shown in Figure 2, which suggests that a low HDL-C is associated directly with poverty in both genders. In particular, in the male group, the HDL categories 1 and 2 were 66.5% of the lowest household income grade 1 (<595 USD). In every income level, the medium low level of HDL-C (40–49 mg/dL) was predominant except for income grade 1, approximately 37.4%, 40.2%, 36.9%, and 42.0% for grades 2, 3, 4, and 5, respectively (Figure 2A). In the female group, the lowest income group 1 showed the largest percentage of the lowest HDL-C category among all income grades and the smallest percentage of the highest HDL-C category around 19.4% and 5.2%, respectively (Figure 2B). Approximately 50% of subjects in the lowest quintile income (group 1) showed an HDL-C level lower than 49 mg/dL, whereas 72% of subjects in the highest quintile of income (group 5) showed an HDL-C level higher than 50 mg/dL. 

The lowest income group had the highest percentage of HDL-C category 1 (33.4% and 19.4% in men and women, respectively) and the highest income group showed the lowest percentage of HDL-C category 1, 22.2% and 7.2% in men and women, respectively (Figure 2). Compared to HDL category 2, HDL category 1 showed a 1.904 fold (*p* < 0.001) higher probability of the lowest income group 1 than the highest income group 5. Compared to the HDL category 3, HDL category 1 showed a 1.963-fold (*p* = 0.001) higher probability of the least income (group 1) than the highest income (group 5). These results strongly suggest that a high income is correlated more closely with a higher HDL-C, particularly in women.

### 3.4. Percentage Ratio of Income Grade in Each HDL-C Category

Interestingly, men and women showed different correlation patterns between income and HDL-C level. Between men and women, there was a significant difference in the household income distribution in the HDL-C category 1 (<40 mg/dL) and the HDL-C category 5 (>70 mg/dL), as shown in Figure 3. In low HDL-C (<40 mg/dL), the male group showed the smallest percentage of income grade 1 (17.5%), whereas the female group showed the largest percentage of income grade 1 (30.5%). In the HDL-C category 5 (>70 mg/dL), 17.8% of the men were in income grade 1, whereas only 7.8% of women were in income grade 1. 

Income grade 1 consisted of 17.5% of the HDL-C category 1 (Figure 3A), which suggests that a low-income grade is associated directly with a low HDL-C level in men. On the other hand, income grade 1 also comprised 17.8% of the highest HDL-C category, which suggests that a higher income level is not associated directly with a higher HDL-C, particularly in the highest HDL category (>70 mg/dL) in men. Furthermore, HDL-C categories 2 (40–49 mg/dL) and 3 (50–59 mg/dL) showed the largest percentage of income grades 4 and 5 (Figure 3A). These results show that a high income is not associated directly with the quantity of HDL-C, which suggests that other factors of HDL might be associated with the quantity of HDL, such as the functionality or quality of HDL particularly in men. 

As shown in Figure 3B, HDL-C category 1 (<40 mg/dL) showed the highest percentage of income grade 1 (30.5%), whereas HDL-C category 5 showed the lowest percentage of income grade 1 (7.8%). In the same context, HDL-C category 1 (<40 mg/dL) had the smallest percentage of income grades 4 (13.5%) and 5 (15.6%) and HDL-C category 5 (>70 mg/dL) showed the largest percentage of income grades 4 (30.7%) and 5 (27.2%). These results suggest that women show a more distinct positive association between income grade and HDL-C level than men.

### 3.5. Average HDL-C Level Depends on the Income Grade

As shown in Figure 4A, in men, the HDL-C level was elevated slightly depending on income from grade 1 (46.4 mg/dL) to 5 (47.4 mg/dL) with significance, according to regression analysis (*p* = 0.01). Although there was no significance among the income grade groups, a Pearson correlation analysis revealed no positive associations between the mean HDL-C level and income grade (*p* = 0.60). In women, the HDL-C level and income grade showed a significant positive correlation (*p* < 0.001), as shown in Figure 4A. The HDL-C level was elevated depending on the increase in income grade from 1 (50.4 mg/dL) to 5 (56.7 mg/dL) with significance (*p* < 0.001) from regression analysis as well as between the income grade groups (*p* < 0.001). As shown in Figure 4B, an increase in income level is positively correlated with the HDL-C category 5 (>70 mg/dL) in women, whereas, in men, an increase in income level is positively correlated with HDL-C until category 3. The difference between HDL-C category from 1 to 5 was found to be significant (*p* < 0.001) in the male group using ANOVA. However, a Pearson correlation analysis revealed that the male group did not show significance (r = 0.03, *p* = 0.66), while the female group showed significant correlations (r = 0.181, *p* < 0.001) between HDL-C and household income grade. Because the five categories of HDL-C were significantly different, the distribution of the income level within each HDL-C category was analyzed. 

To confirm the results of Figure 4, analysis of covariance (ANCOVA) was carried out after adjusting some variables. Supplementary Table S1 and Supplementary Figure S3 also show the positive direct relationship between the household income grade and HDL-C levels. The multivariate adjusted means of the total serum HDL-C levels (SEM) were 49.397 (0.407), 50.320 (0.360), 51.235 (0.334), 51.985 (0.328), and 51.858 (0.317) mg/dL, for household income grades 1, 2, 3, 4, and 5, respectively, after adjusting for gender, frequency of diabetes, use of anti-dyslipidemic drug, myocardial infarction or angina pectoris, physical exercise, alcohol intakes, and smoking habit (Supplementary Figure S3). These positive relationships between household income and HDL-C levels were similar to the result with Figure 4 and Supplementary Figure S1, which were not adjusted with the variables. The test statistic F for HDL-C was F = 6.159 with an increasing household income group after adjusting for the same variables (*p* < 0.001) (Supplementary Table S1).

### 3.6. Average Household Income Grade Depends on the HDL-C Category

The HDL-C adjusted average income showed that women were poorer (average income grade 3.15) than men (average income grade 3.30) in the low HDL category, as shown in Figure 4B. Regression analysis revealed both groups to have a significant increase in HDL-C with income, but women showed a higher significance (*p* < 0.001) than men (*p* = 0.03). In men, the HDL-C-adjusted average income showed that the HDL category 3 (50–59 mg/dL) had the highest average income (3.43, *p* = 0.00007) and HDL category 5 (>70 mg/dL) showed the third highest average income (3.21). In women, however, the HDL-C adjusted average income increased gradually from 2.61 in the HDL-C category 1 to 3.58 in the HDL-C category 5, as shown in Figure 4B. Women showed a proportional increase in the HDL-C level with increasing average income, which suggests that the mean income grade is also associated directly with the HDL-C level. Overall, men and women showed different correlations between the HDL-C adjusted average income grades. 

### 3.7. Incidence of Dementia and HDL-C Based on Age

As shown in Figure 5A, women showed a sharp decrease in HDL-C with age particularly in those older than 50 years, whereas men showed a more stable and smaller decrease in HDL-C with age. Men and women in their 80s showed a similar HDL-C level: 45.9 ± 10.9 and 46.6 ± 10.9, respectively. This suggests that menopause in women in their 50s might influence the sharp decrease in HDL-C. Data from the Korean Health Bigdata Hub showed that the incidence of dementia was 459,421 patients in 2017 (131,025 men and 328,396 women), which suggests that 71.4% of patents were women (Figure 5B). There were more male patients than females aged 40–49 years with 488 male and 404 female patients. On the other hand, female patients overtook the male patients after the age of 50 years (3118 men and 3698 women). Furthermore, in patients older than 60, women (n = 19,659) showed a 1.3-fold higher number of cases than men (n = 15,259). The difference became much larger in those in their 70s and 80s. Women showed a 1.9-fold and 3.3-fold higher incidence of dementia than men, respectively. There were 1.94 times more female dementia cases (n = 103,623) than male patients (n = 53,355) in their 70s, and 3.3 times more female patients (n = 211,580) than male patients (n = 63,513) in their 80s. 

## 4. Discussion

This study revealed a positive correlation between the household income grade and HDL-C level. A low HDL-C level is associated directly with a low household income in an age-dependent manner. To the best of the authors’ knowledge, the current finding is the first report to show the distribution of HDL-C based on the household income grade in the recent Korean population. A low HDL-C level (>39 mg/dL) is linked directly to a low-income grade in men and women. In particular, women showed a larger decrease in HDL-C as increase of age and decrease of income. The HDL-C adjusted average income showed that the low HDL group had the lowest income (grade 3.12) and the medium HDL group had the highest income (3.43, *p* = 0.00007). In women, the HDL-C adjusted average income showed that the low HDL group had the lowest income (grade 2.61) and the highest HDL group had the highest income (3.58, *p* < 0.001). Women showed a straightforward increase in income level with the HDL-C level.

A population with a lower socioeconomic status is at higher risk of diabetes [22] and dyslipidemia [23], as well as MCI and dementia [24], particularly in older adults [25]. The characteristics of the patients with mild cognitive impairment (MCI) are a low HDL-C [26] and high serum amyloid A [27]. On the other hand, the atherogenic index (AI) is the most reliable indicator of the cardiovascular disease risk. The AI index was calculated using the total cholesterol (TC)/high-density lipoprotein cholesterol (HDL-C) ratio [28]. There was no difference in the serum TC and LDL-C in patients with MCI compared to the control [9]. Although the total 5535 subjects in the current study exhibited normal TC, HDL-C, LDL-C, and TG levels, the distribution of TC and HDL-C differed according to gender and age.

Many clinical trials have failed to show an effective treatment for Alzheimer’s disease despite the decades of promising research [29]. The HDL-C and incidence of dementia is inextricably linked because apolipoprotein A-I (apoA-I) can cross the blood brain barrier (BBB) to form apoA-I-HDL [30]. The HDL in the brain can bind with Aβ and remove cerebrospinal fluid. The incidence of dementia is also associated intimately with a decrease in the HDL-C level in those older than 50 years. In particular, women showed a considerable increase in dementia after 60 years. In the same context, a recent study on the Japanese population showed that high midlife HDL-C levels are inversely associated with both late-life MCI and dementia [31]. Other studies with the American population also showed that higher HDL cholesterol levels are associated with better cognitive function [32]. After menopause, women showed a high risk of CVD similar to men [33] because the protection effect of estrogen and high HDL-C disappeared. The current result strongly suggests that a high HDL-C level also protects women from the incidence of dementia (Figure 5), as in the previous review [34] because the risk factors of CVD play a critical role in the etiology of dementia. Other groups also reported close associations between low HDL-C levels and cognitive impairment in older adults [35].

This suggests that women have a stronger direct association of poverty with low HDL-C levels than men. Further studies will be needed to compare the quality of women’s HDL after menopause. There might be a functional difference in HDL between men and women in later-life depending on the significant difference in the incidence of dementia. Women with a low income and those in minority groups in the American population have some of the highest rates of obesity, diabetes, and hypertension [7]. 

Many prospective studies showed that the serum HDL-C level declined with age [36]. Although there are conflicting data regarding the change in HDL-C with age, the HDL-C in adults tended to decrease with age in both men and women in prospective studies [37,38]. Additionally, it has been reported that the HDL-cholesterol level was significantly decreased in post-menopausal women [39]. The calculated atherogenic index (Total Cholesterol/HDL ratio) was significantly increased in post-menopausal women as compared to that in premenopausal women [40]. The other previous study also reported the decrease of HDL-C along with age. The Rancho Bernardo Study 1984–1994 showed that HDL-C levels decreased in older men and women with an increase in age [41]. Our current finding aligns with the Rancho Bernardo study. 

Several lifestyles, such as exercise and dietary habits, are critical factors affecting the serum HDL-C level. Performing regular exercise is important for raising the HDL-cholesterol and its functionality [42]. On the other hand, lower socioeconomic groups are less likely to perform regular exercise due to financial constraints. In addition, the lower socioeconomic groups are more likely to consume more fast foods (or junk foods) and unhealthy food ingredients that are enriched with fructose corn syrup, saturated fat, and trans-fatty acids. The consumption of more unhealthy foods is associated with a lower HDL-C level and higher incidence of metabolic syndrome [43]. Concerning the intake of various food groups, low socioeconomic groups prefer white bread, potatoes, and pasta or rice and refined cereals compared to those of high socioeconomic groups [44]. The high socioeconomic groups prefer whole bread or wholegrain products that have a lower glycemic index [45]. Overall, wealthy people are more likely to have higher HDL-C levels than those who are less prosperous because they exercise more and consume healthier food.

In summary, men and women showed a direct association of income grade and HDL-C level, even though there was a slightly different correlation. Men in the lowest income group had the highest percentage of HDL-C category 1 (33.4%) and the highest income group showed the lowest percentage of HDL-C category 1 (22.2%). In contrast, women in the HDL category 1 had the highest percentage of the lowest income grade (30.5%). HDL category 5 contained the smallest percentage of the lowest income level (7.8%). In conclusion, in both genders, the lower income group showed a higher prevalence of low-HDL-C levels. The HDL-C levels decreased gradually and dramatically in those older than 50 years, and the incidence of dementia increased considerably in those in their 60s.

## 5. Conclusions

In both the male and female groups, the lower income group showed a larger prevalence of low-HDL-C levels. In particular, in the female group, the HDL-C level decreased sharply after 50 years of age likely due to menopause. The decrease in HDL-C after middle age was strongly associated with the considerable increase in dementia in later life. 

## Figures and Tables

**Table 1:** General characteristics of study participants.

Characteristics	Men *N* = 2469			Women *N* = 3066			* *p*-Value	Total *N* = 5535		
		Mean ± SD			Mean ± SD				Mean ± SD	
Age (years)		51.3 ± 16.4			51.9 ± 16.3		0.147		51.6 ± 16.4	
TC (mg/dL)		191.3 ± 38.0			194.9 ± 38.1		0.0004		193.3 ± 38.1	
TG (mg/dL)		158.8 ± 126.1			113.4 ± 74.8		<0.001		133.6 ± 103.4	
HDL-C (mg/dL)		47.2 ± 11.1			54.4 ± 12.4		<0.001		51.2 ± 12.4	
HDL-C category	N (%)		Age	N (%)		Age		N (%)		Age
<40	628 (25.4)	34.8 ± 3.8	54.3 ± 15.8	315 (10.3)	35.3 ± 3.5	61.3 ± 14.9	0.028	943 (17.0)	35.0 ± 3.7	56.6 ± 15.8
40–49	950 (38.5)	44.5 ± 2.8	50.4 ± 16.3	814 (26.5)	45.0 ± 2.8	54.5 ± 16.1	0.0003	1764 (31.9)	44.7 ± 2.8	52.3 ± 16.3
50–59	584 (23.7)	53.9 ± 2.8	50.2 ± 16.6	988 (32.3)	54.4 ± 2.8	50.8 ± 16.1	0.002	1572 (28.4)	54.2 ± 2.8	50.6 ± 16.3
60–69	206 (8.3)	63.3 ± 2.7	49.7 ± 17.1	614 (20.0)	64.0 ± 2.9	48.6 ± 16.3	0.009	820 (14.8)	63.8 ± 2.9	48.9 ± 16.5
>70	101 (4.1)	77.5 ± 7.6	49.5 ± 17.6	335 (10.9)	78.1 ± 8.0	45.8 ± 14.0	0.499	436 (7.9)	77.9 ± 7.9	46.7 ± 15.0
Glucose		104.3 ± 26.6			98.8 ± 22.7		<0.001		101.2 ± 24.7	
Income grade		3.3 ± 1.4			3.15 ± 1.4		0.00006		3.22 ± 1.4	
1	329 (13.4)	1	63.2 ± 17.0	496 (16.2)	1	67.9 ± 12.7		825 (14.9)	1	66.0 ± 14.7
2	433 (17.5)	2	56.9 ± 16.7	575 (18.8)	2	54.9 ± 16.2		1008 (18.2)	2	55.8 ± 16.4
3	512 (20.7)	3	49.4 ± 15.9	652 (21.3)	3	48.9 ± 15.4		1164 (21.0)	3	49.1 ± 15.6
4	561 (22.7)	4	46.9 ± 14.2	663 (21.5)	4	46.1 ± 14.1		1224 (22.1)	4	46.4 ± 14.1
5	634 (25.7)	5	46.6 ± 14.0	680 (22.2)	5	46.1 ± 13.2		1314 (23.8)	5	46.3 ± 13.6

Data are expressed as mean ± SD (standard deviation) or N (%). * *p* value for difference between men and women (*p* < 0.05). TC, total cholesterol. TG, triglycerides. HDL-C, high-density lipoprotein-cholesterol. %HDL-C, (HDL-C/TC) * 100.

**Table 2:** Distribution of age and income grade depends on HDL-C. Data are expressed as mean ± SD (standard deviation) or N (%). * *p* value for difference between men and women (*p* < 0.05).

Characteristics	Men *N* = 2469		Women *N* = 3066		* *p*-Value	Total *N* = 5535	
	*N* (%)	Mean ± SD	*N* (%)	Mean ± SD		*N* (%)	Mean ± SD
Age							
20–29	300 (12.2)	49.9 ± 11.1	327 (10.7)	58.1 ± 11.3	<0.001	627 (11.3)	54.2 ± 11.9
30–39	376 (15.2)	47.2 ±10.2	460 (15.0)	57.9 ± 12.4	<0.001	836 (15.1)	53.1 ± 12.7
40–49	454 (18.4)	47.3 ±10.6	585 (19.1)	56.0 ± 12.6	<0.001	1039 (18.8)	52.2 ± 12.5
50–59	502 (20.3)	46.8 ±11.2	617 (20.1)	55.2 ± 12.4	<0.001	1119 (20.2)	51.4 ± 12.6
60–69	435 (17.6)	47.0 ±11.6	564 (18.4)	51.7 ± 12.0	<0.001	999 (18.0)	49.6 ± 12.0
70–79	320 (13.0)	45.7 ±11.7	387 (12.6)	50.2 ± 11.4	<0.001	707 (12.8)	48.1 ± 11.8
<80	82 (3.3)	45.9 ± 10.9	126 (4.1)	46.6 ± 10.9	0.656	208 (3.8)	46.3 ± 10.9
Income grade							
1	329 (13.3)	46.4 ± 12.8	496 (16.2)	50.4 ± 12.0	0.000006	825 (14.9)	48.8 ± 12.5
2	433 (17.5)	46.9 ± 11.7	575 (18.8)	52.6 ± 11.7	<0.001	1008 (18.2)	50.1 ± 12.0
3	512 (20.7)	47.2 ± 10.8	652 (21.3)	54.6 ± 12.3	<0.001	1164 (21.0)	51.4 ± 12.2
4	561 (22.7)	47.6 ± 10.3	663 (21.6)	56.5 ± 12.8	<0.001	1224 (22.1)	52.4 ± 12.5
5	634 (25.8)	47.4 ± 10.7	680 (22.1)	56.7 ± 12.3	<0.001	1314 (23.8)	52.2 ± 12.4

Data are expressed as mean ± SD (standard deviation) or N (%). * *p* value for the difference between men and women (*p* < 0.05).

## Supplementary Materials

The following are available online at https://www.mdpi.com/1660-4601/16/18/3329/s1. Figure S1: Pearson’s correlation analysis of HDL-C and household income grade in men (A) and women (B). Regression analysis was performed to find a correlation coefficient between HDL-C and household income grade. For men group showed r = 0.026 (*p* = 0.173) and women group showed r = 0.164 (*p* < 0.001). Figure S2: Pearson’s correlation analysis of HDL-C and age in men (A) and women (B). Regression analysis was performed to find a correlation coefficient between HDL-C and age. For men group showed r = 0.085 (*p* = 0.00001) and women group showed r = 0.240 (*p* < 0.001). Figure S3: Positive direct relationships between the HDL-C level in according to household income by analysis of covariance (ANCOVA), after adjusting for age, frequency of diabetes, use of anti-dyslipidemic drug, myocardial infarction or angina pectoris, physical exercise, alcohol intakes, and smoking habit. Bars represent the standard errors of the mean (SEM). The x-axis is household income divided into five groups. The y-axis is HDL-C level (mg/dL). Table S1: Positive direct relationships between the HDL-C level and household income by analysis of covariance (ANCOVA).
