# Supplementary figures and images for: Decrease in HDL-C is Associated with Age and Household Income in Adults from the Korean National Health and Nutrition Examination Survey 2017: Correlation Analysis of Low HDL-C and Poverty
Source: Int J Environ Res Public Health. 2019 Sep 10;16(18):3329. doi: 10.3390/ijerph16183329 (PMC6765955; doi:10.3390/ijerph16183329)

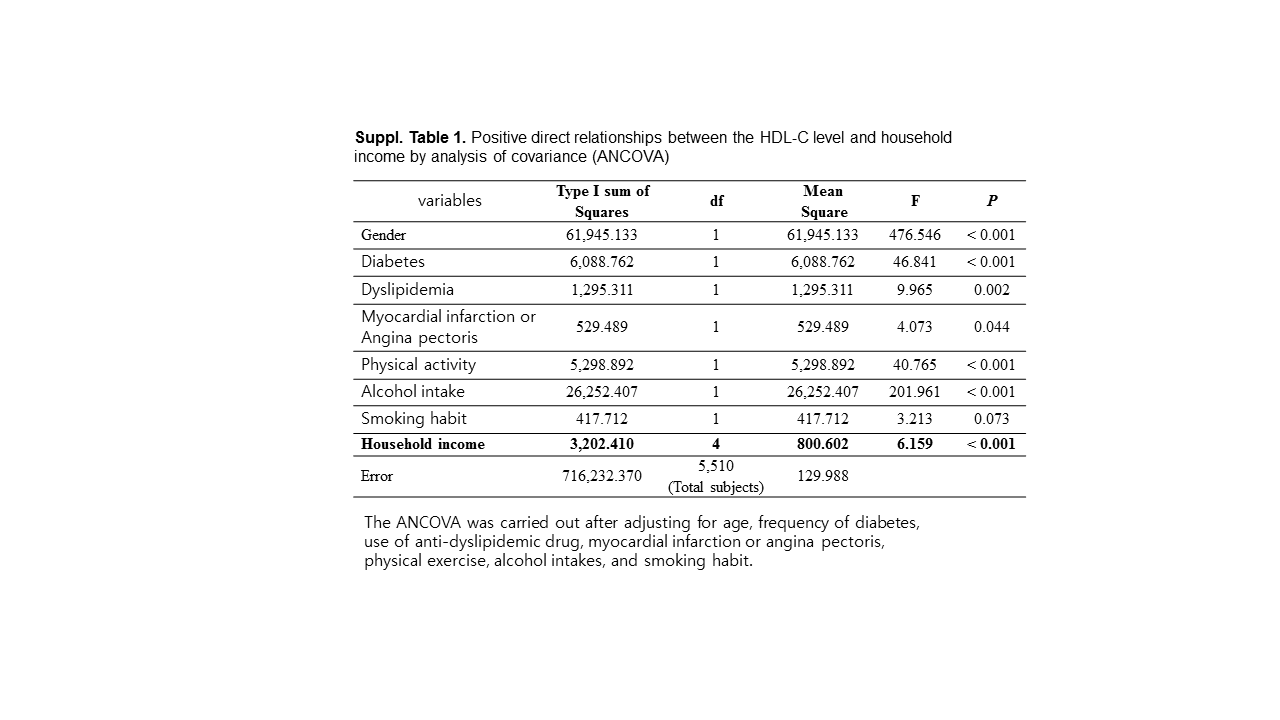

Supplement: Supplementary file 1 [file ijerph-16-03329-s001.zip › 6. Suppl. Table 1..TIF]

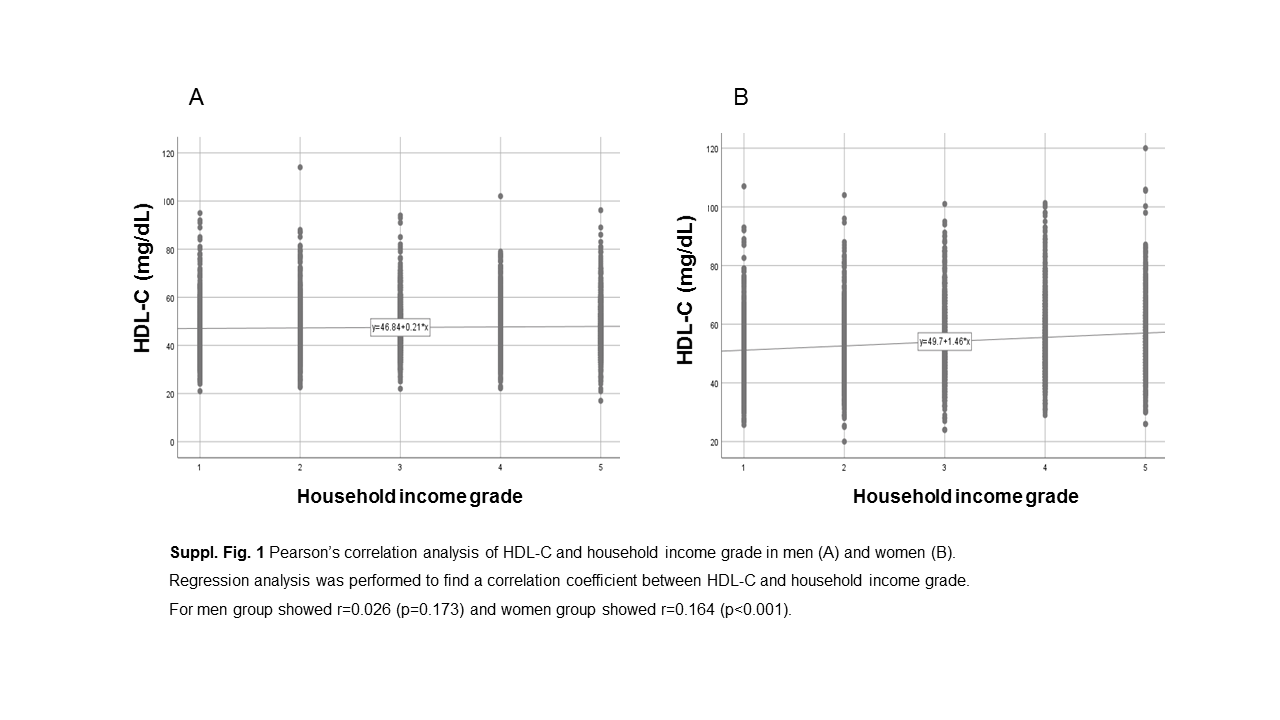

Supplement: Supplementary file 1 [file ijerph-16-03329-s001.zip › 7. Suppl. Fig. 1..TIF]

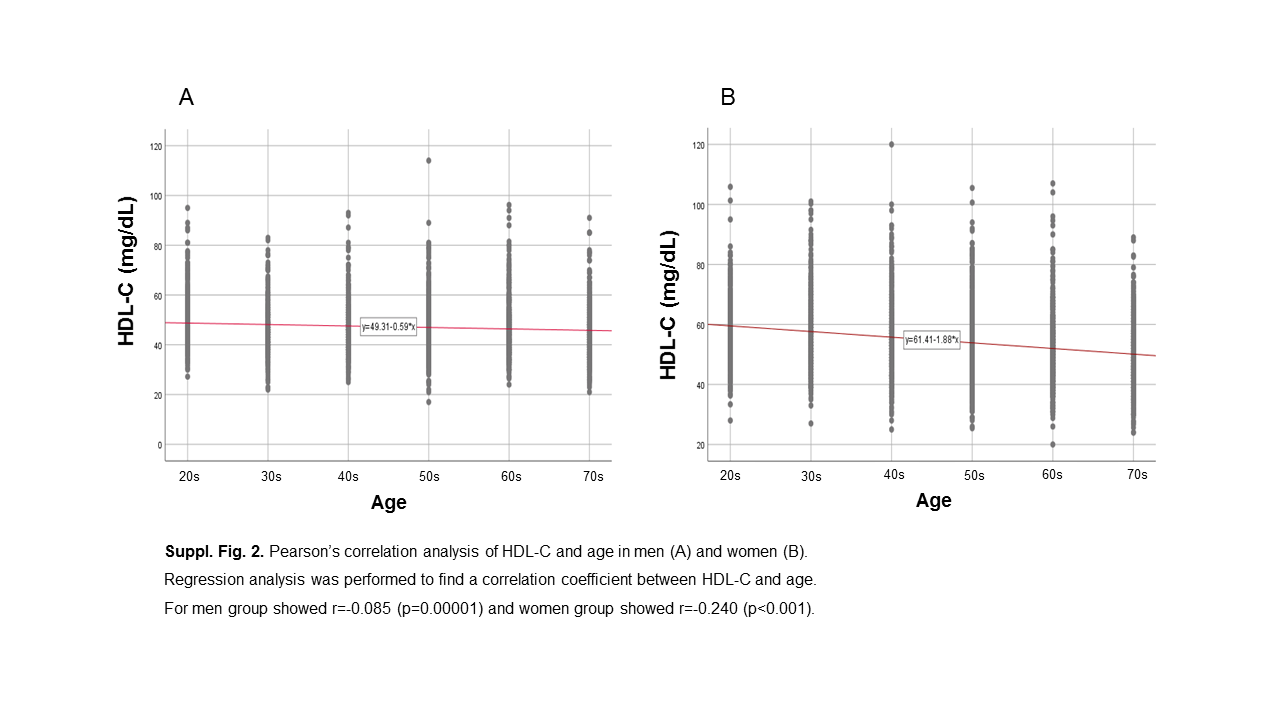

Supplement: Supplementary file 1 [file ijerph-16-03329-s001.zip › 8. Suppl. Fig. 2..TIF]

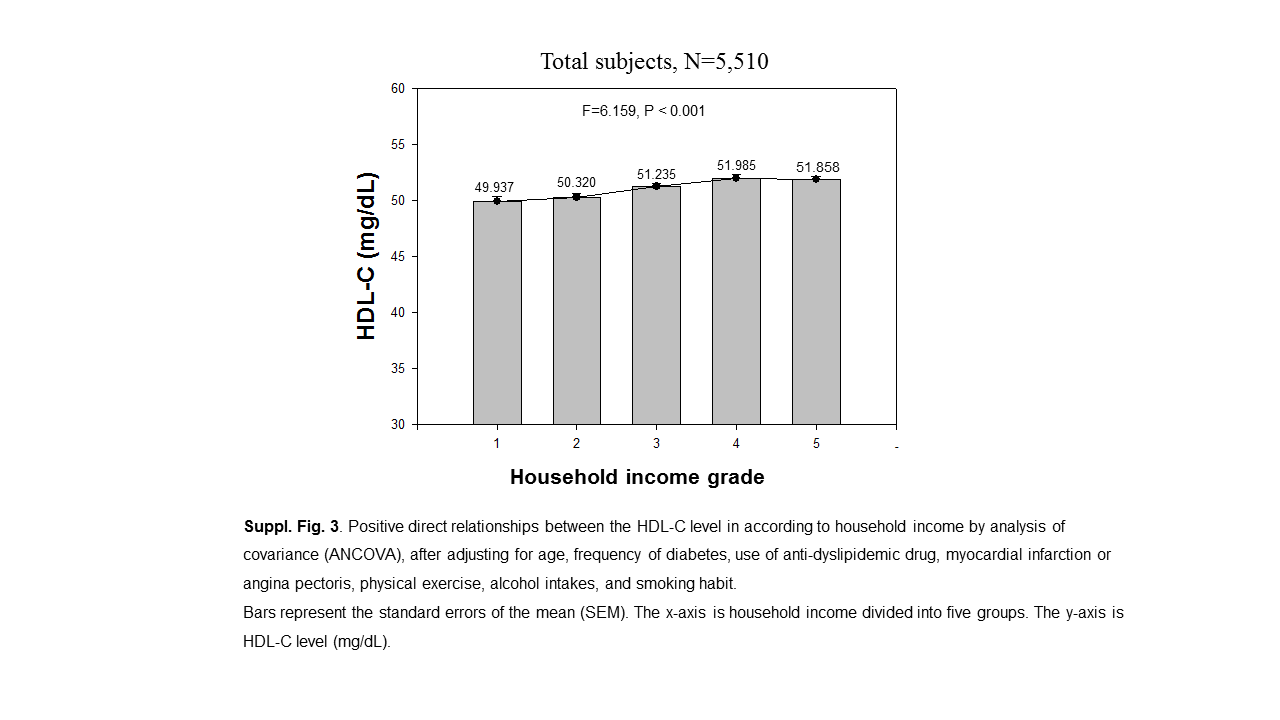

Supplement: Supplementary file 1 [file ijerph-16-03329-s001.zip › 9. Suppl. Fig. 3..TIF]
